# The enhanced information flow from visual cortex to frontal area facilitates SSVEP response: evidence from model-driven and data-driven causality analysis

**DOI:** 10.1038/srep14765

**Published:** 2015-10-05

**Authors:** Fali Li, Yin Tian, Yangsong Zhang, Kan Qiu, Chunyang Tian, Wei Jing, Tiejun Liu, Yang Xia, Daqing Guo, Dezhong Yao, Peng Xu

**Affiliations:** 1 Key Laboratory for NeuroInformation of Ministry of Education, School of Life Science and Technology, University of Electronic Science and Technology of China, Chengdu, 610054, China; 2 Center for Information in BioMedicine, University of Electronic Science and Technology of China, Chengdu, 610054, China; 3 School of Computer Science and Technology, Southwest University of Science and Technology, Mianyang, 621010, China; 4 College of Bio-information, ChongQing University of Posts and Telecommunications, ChongQing, 400065, China

## Abstract

The neural mechanism of steady-state visual evoked potentials (SSVEP) is still not clearly understood. Especially, only certain frequency stimuli can evoke SSVEP. Our previous network study reveals that 8 Hz stimulus that can evoke strong SSVEP response shows the enhanced linkage strength between frontal and visual cortex. To further probe the directed information flow between the two cortex areas for various frequency stimuli, this paper develops a causality analysis based on the inversion of double columns model using particle swarm optimization (PSO) to characterize the directed information flow between visual and frontal cortices with the intracranial rat electroencephalograph (EEG). The estimated model parameters demonstrate that the 8 Hz stimulus shows the enhanced directional information flow from visual cortex to frontal lobe facilitates SSVEP response, which may account for the strong SSVEP response for 8 Hz stimulus. Furthermore, the similar finding is replicated by data-driven causality analysis. The inversion of neural mass model proposed in this study may be helpful to provide the new causality analysis to link the physiological model and the observed datasets in neuroscience and clinical researches.

When circular stimulus with frequency distributed in certain range is presented to the subject, the oscillatory wave responding to the stimuli frequency can be observed in the occipital area, which is called steady-state visual evoked potential (SSVEP)^1^. Various studies have revealed that only the stimulus with a low frequency, especially in the range below 30 Hz, can effectively evoke a strong SSVEP response^1^,^2^,^3^. Because SSVEP has the high signal-to-noise (SNR), it has been widely used in brain-computer interface (BCI), visual attention, binocular rivalry, and working memory^2^,^4^,^5^,^6^,^7^,^8^. The studies based on electroencephalograph (EEG), magnetoencephalograph (MEG), and functional magnetic resonance imaging (fMRI) consistently reveal that the SSVEP response is widely distributed over the occipital and the other areas, including parietal, temporal, frontal, and prefrontal lobes^4^,^9^,^10^,^11^,^12^,^13^. Driven from the involvement of multiple brain areas in SSVEP, several studies have probed the information linkages among those regions in their network analysis using fMRI, scalp EEG, and intracranial EEG data^12^,^13^,^14^,^15^,^16^. Especially, our previous studies based on intracranial EEG of rat and human scalp EEG using the undirected network analysis method (i.e., coherence) both demonstrate that SSVEP generation is closely correlated with strong linkages between frontal and occipital lobes^14^,^17^. However, because of the utilization of the undirected network analysis, it is impossible to reveal the directed information exchange between frontal and visual cortices, i.e., which directed information flow enhances the interaction between the two brain areas. Actually, there exists several directed network analysis methods including Granger causality (GC)^18^,^19^, partial directed coherence (PDC)^20^, and directed transfer function (DTF)^15^,^21^ that can delineate the directed information flow across the concerned brain areas. Keil *et al*. applied the GC analysis to investigate the re-entrant modulation of visual cortex in the affective processing. They found the enhanced influence from anterior cortical to visual cortical sites during the subjects’ viewing emotionally arousing content, and this indicated that re-entrant modulation of visual system is enhanced as a function of the emotional arousal of the visual scene^19^. However, those data-driven methods are mainly derived from the mathematical aspect without specific consideration for the physiological basis^22^,^23^,^24^.

Recently, due to the biophysical derivations, the physiological models attract wide attention in neuroscience, among which the neural mass model and its corresponding variants are mostly used in various studies^22^,^25^,^26^,^27^,^28^. The goal of neural mass model is to understand the neuronal architectures that generate electrophysiological data^28^. Neural mass model is firstly introduced by Da Silva *et al*.^26^. In these models, the dynamics of entire neural populations and their synapses are described using just a few state variables (i.e., a few differential equations) under the assumption that neurons in the same population share similar inputs and synchronize their activity. Besides a smaller computational complexity, these models offer a more parsimonious description of neural dynamics in terms of parameters and mechanisms involved, generally ascribing rhythm generation to feedback loops between excitatory and inhibitory neural populations. Jansen *et al*. improved the neural mass models^29^ by encompassing the interaction between three neural populations with different synaptic kinetics (pyramidal neurons, excitatory interneurons, and inhibitory interneurons). In 1995, Jansen and Rit further developed the coupled double column model to generate visual evoked potential using single column to simulate the visual cortex and frontal cortex, respectively^30^. The simulation study revealed that those visual components like P1, N1 could be observed by adjusting the intercolumn connectivity coefficients. Another study by Spiegler *et al*. also proved that neural mass model was indeed able to explain frequency entrainment that was observable during a photic driving experiment^31^.

Though many efforts were made for both recording and analysis aspects, neural mechanisms of SSVEP are still unclear and need to be deeply explored. Moreover, the model based analysis is less involved in the SSVEP related studies, which may provide new insights to reveal SSVEP mechanism. In the current work, derived from the reported successful application of double columns model for visual evoked potential, we utilized this model to simulate the SSVEP response for different external circular stimuli and those model parameters were optimized using particle swarm optimization (PSO) to fit the actual intracranial EEG recordings of rats. Then based on the estimated model parameters from the data of different frequency conditions, the directed information flow between frontal and visual cortices can be revealed. The conducted study may find the underlying differences of information exchange for those frequency stimuli that can evoke SSVEP or not from the physiological model perspective.

## Results

### Double column model based information transfer

**Figure 1:**
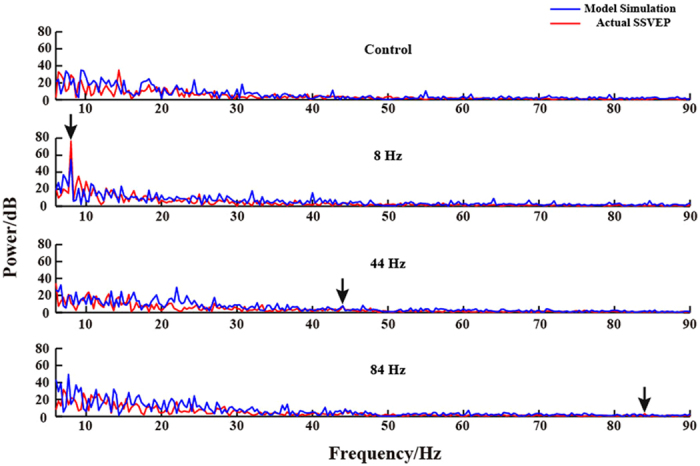
The power spectrum of the actual EEG signal and the simulated data for the four conditions of one rat. The red line indicates the double column model simulated signal and the blue line indicates the actual SSVEP signal.

Based on the PSO, the parameters (C_1_, C_2_, K_1_, and K_2_) were optimally adjusted to fit the recorded EEGs both in occipital and frontal areas. For all the estimated models, the relative errors were below 0.01, and the time series were well fitted. Figure 1 gives the corresponding power spectrum for control, 8 Hz, 44 Hz, and 84 Hz stimuli of one rat derived from the output waveform of Column 1 (i.e., visual cortex) and the actual EEG signal of V1_L electrode.

As shown in Fig. 1, the power spectrum between the simulated and actual EEG depicts the similar pattern, and the important aspect is that the simulated responses for 8 Hz stimulus reveal the obvious SSVEP response at the visual cortex, while other stimuli do not show the strong responses at the concerned frequency points, which is consistent with the response reflected by the actual EEG.

**Figure 2:**
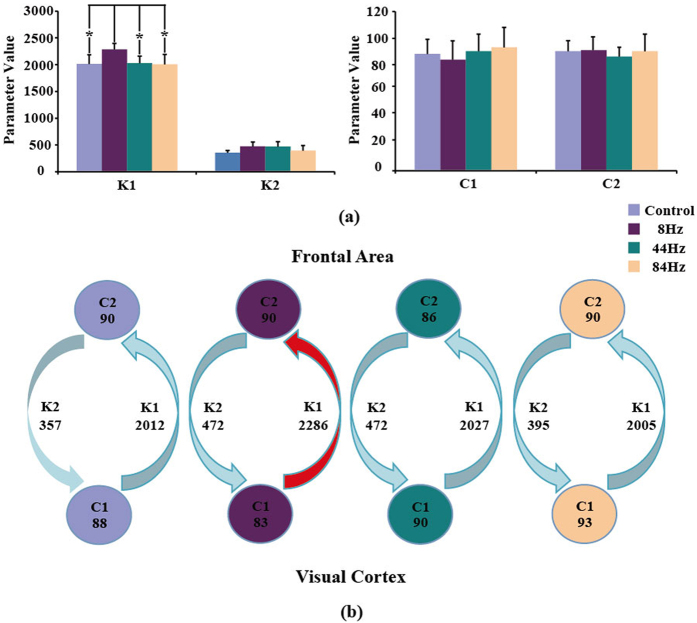
The double column model parameter (K_1_, K_2_, C_1_, and C_2_) differences among the four conditions. (**a**) Statistical difference; (**b**) The corresponding information exchange between the Column 1 (occipital lobe) and Column 2 (frontal cortex). K_1_ indicates the information exchange from Column 1 to Column 2, K_2_ indicates the information exchange from Column 2 to Column 1, C_1_ represents the information exchange inside Column 1, and C_2_ represents the information exchange inside Column 2.

The estimated model parameters (C_1_, C_2_, K_1_, and K_2_) were averaged across 10 rats for each of the four conditions. Figure 2(a) listed the averaged four parameters corresponding to the four states. Then, the one-way repeated-measure ANOVA with Frequency (8 Hz vs. 44 Hz vs. 84 Hz vs. Control) was used to test the properties of double column model based information transfer. Figure 2(a) showed that the significant main effect of frequency occurs in K_1_ [F(3, 27) = 7.417, p = 0.009, Greenhouse-Geisser correction]. Post-hoc test (paired t-test) showed that K_1_ of 8 Hz stimulus were statistically larger than that of the other three conditions (For 44 Hz: p = 0.000, 84 Hz: p = 0.008, and control: p = 0.010, Bonferroni correction) While, no statistical differences were found for C_1_, C_2_, and K_2_ across the four conditions. Based on the estimated four parameters, the directed information exchanges between the two columns were summarized as below in Fig. 2(b). Figure 2(b) clearly showed that the main difference among the four conditions was that 8 Hz stimulus revealed the strong information flow from occipital to frontal area compared to other three conditions. At the same time, 8 Hz stimulus also exhibited the largest overall information exchange (calculated as K_1_ + K_2_) between the two columns.

### PDC based information transfer pattern

**Figure 3:**
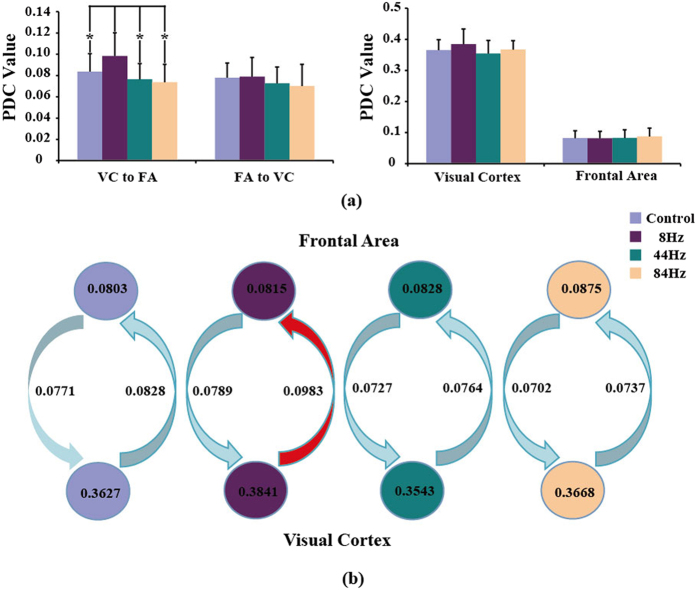
The directed information flow differences among the four conditions revealed by PDC analysis. (**a**) Statistical difference; (**b**) The corresponding information exchange between the visual cortex and frontal area. Visual cortex (VC) contains the four electrodes V1_L, V1_R, V2_L, and V2_R; frontal area (FA) contains the two electrodes FrA_L and FrA_R. And VC to FA indicates the directed information exchange from visual cortex to frontal area, FA to VC indicates the directed information exchange from frontal area to visual cortex, Visual Cortex represents internal information flows of visual cortex, Frontal area represents the internal information flows of frontal area.

For each rat, PDC analysis was applied to generate the corresponding directed flow linkage strengths. The directed flow linkage strengths were then averaged across ten rats for each of the four conditions. One-way repeated-measure ANOVA with Frequency (8 Hz vs. 44 Hz vs. 84 Hz vs. Control) was also used to investigate the difference of flow strength existing in the four conditions. PDC analysis revealed the similar directed information flow as that demonstrated by double column model. Specifically, the significant main effects of frequency were observed in the information flows from visual cortex to frontal area [F(3, 27) = 11.976, p = 0.000, Greenhouse-Geisser correction], Then, post-hoc test (paired t-test) showed that 8 Hz stimulus had the statistically larger information flow from visual cortex to frontal area than that of the other three conditions (For 44 Hz: p = 0.003, 84 Hz: p = 0.005, and control: p = 0.041, Bonferroni correction). No statistical differences were observed for the feedback flow from frontal area to visual cortex, the internal linkages within visual cortex and frontal area (Fig. 3(a)). Based on the PDC flow strengths, the information exchanges between the two brain areas were given in Fig. 3(b). Figure 3(b) also showed that 8 Hz stimulus exhibits the strongest overall information between frontal area and visual cortex.

## Discussion

Occipital and frontal areas play important roles for the SSVEP generation. Our previous studies based on both intracranial and scalp EEG recording consistently revealed that the frequency stimulus that can evoke strong SSVEP response will correspond to a denser brain network especially with much stronger linkages between frontal and occipital areas^14^,^16^. Because our previous studies are based on the undirected network analysis, it cannot uncover the directed information flow, i.e., which direction of flow dominately contributes to the linkage enhancement. To quantitatively discover the information exchange between the two areas, in current work, we adopted two different causality analysis methods. One is based on the double column model, and another is based on PDC analysis.

As shown in Fig. 1, similar to the actual EEG recordings, only the output of double column model for 8 Hz stimulus has the related strong SSVEP response. K_1_ and K_2_ delineate the information exchange between visual cortex and frontal area, while C_1_ and C_2_ represent the internal linkage for visual cortex and frontal area. The detailed parameters in Fig. 2(a) show that K_1_ of 8 Hz stimulus is statistically larger than the other three conditions (*p* < 0.05), while K_2_, C_1_, and C_2_ do not exhibit the obvious difference for the four conditions, which indicates that the generation of SSVEP is mainly due to the strong information transfer from visual cortex to frontal area. Consistent with the information flow revealed by the double column model, PDC analysis also proves that the strongest information flow from visual cortex to frontal area is observed for 8 Hz stimulus (*p* < 0.05) among the four conditions, and other three concerned flow linkages are not of obvious difference for the four conditions.

In essence, double column model is derived from the anatomical information, and it may reflect the more actual physiological basis. While PDC is essentially derived from the mathematical assumption without referring to the physiological information^20^. Though the differences of working mechanism for the two analysis approaches do exist, the results achieved from them are still consistent. They both indicate that when different stimuli are applied, the information exchange within the corresponding sub-networks (i.e., visual cortex and frontal area) has no obvious difference, and the information feedback from frontal area to visual cortex is also kept relatively stable for the four conditions. After visual cortex receive visual information input, visual information will be delivered to frontal area for further processing. As SSVEP response can be regarded as the signal enhancement or transfer of the external stimuli in brain, it is reasonable to assume that if a stimulus can evoke the corresponding SSVEP response, the corresponding response network should have powerful processing and stable transferring ability to keep the information of the flickering stimuli as intact as possible. Various studies from fMRI, EEG, and MEG have revealed that frontal lobe is highly involved in SSVEP processing^4^,^32^,^33^. Therefore, we assume that the strong information flow from visual cortex to frontal area for 8 Hz stimulus may guarantee the stimulus information is intactly delivered to frontal area for further processing, which may account for the finding that the 8 Hz response is observed to be distributed over the whole brain including occipital and frontal areas when intracranial EEG recording is used.

Another aspect revealed the two analyses is that when the overall information between frontal and occipital cortices is considered, 8 Hz stimulus shows the strongest information exchange between these two concerned brain regions, which is consistent with the previous finding based on both scalp EEG of human and intracranial EEG of rats^14^,^16^. In other words, the information increase of 8 Hz stimulus is mainly attributed to the enhanced information transfer from visual cortex to frontal area, that is, the information transfer strength from visual cortex to frontal area determines whether the stimulus could evoke the corresponding SSVEP response or not. However, it needs to point out that PDC based analysis only considers the direct connections between the frontal area and visual cortex, and information exchange may be indirectly transferred through other nodes like Pt_A, M1. Therefore, the PDC based analysis may miss some information flow.

As revealed in our previous studies, the individual differences existed among the subjects^14^,^16^. In current study, our analyses mainly focused on the averaged group level. It is worthy to explore the individual differences with the method presented here. We will further to explore these phenomena in the future studies. As summarized in previous studies^9^,^13^,^34^, there are multiple brain areas involved for SSVEP generation. The current work mainly focuses on the information exchange between frontal area and occipital. In the future, we will construct the bigger neural mass network model to include more sources, such as medial occipital and mid-temporal regions to explore the mechanism of SSVEP. A possible limitation of current study is that the unipolar electrodes are used to record LFP based on the common reference placed at Cerebrum. Just as proved in Bollimunta *et al*.^35^, unipolar recording may provide the susceptible linkages for causality analysis and other more reliable neural signal types like biopolar recording and multiunit activity (MUA) need to be considered in the future work. Furthermore, we will also resort to the multimodalities (EEG and fMRI) to replicate the results on the human subject, and add the cognitive tasks in the experiments to explore the related brain mechanisms, such as the visual attention using the SSVEP as the frequency tags.

## Methods

All experimental protocols were performed in accordance with the Ethical Committee on Animal Experimentation of University of Electronic Science and Technology of China (UESTC). And the performed methods were also in accordance with the guidelines approved by the institutional review board of the Key Laboratory of NeuroInformation of Ministry of Education at UESTC.

### Materials

**Figure 4:**
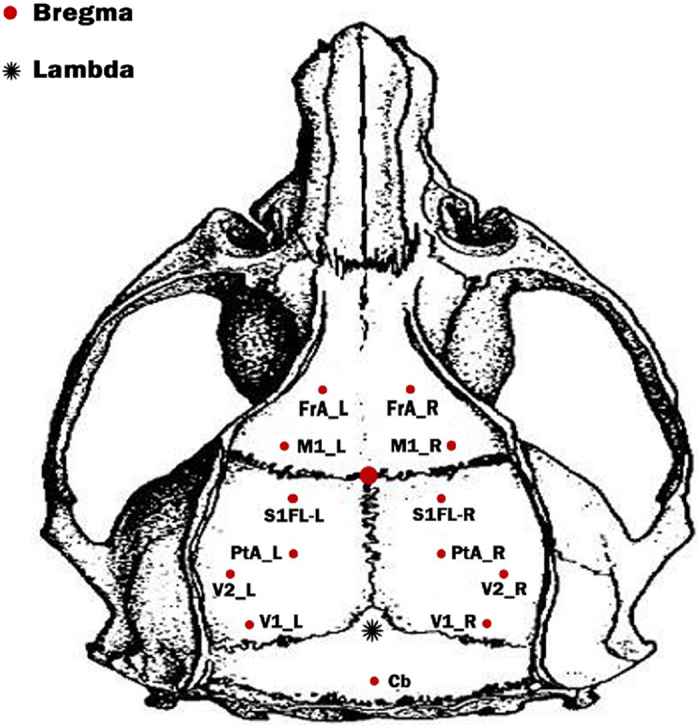
The spatial distribution of 13 intracranial electrodes that were used for recording the intracranial SSVEP signal. During the data recording, the cerebrum (Cb) serves as the reference.

Ten male Wistar rats (body weight 290–320 g) were included in the study. Electrode implantation was performed under general anesthesia (sodium pentobarbital 60 mg/kg bodyweight, i.p.), complemented with 0.6 ml atropine sulfate (0.5 mg/ml, s.c.) to prevent excessive secretion. During stereotactic surgery, wounds were infiltrated with lignocaine (2%). Additional pentobarbital (15 mg/kg) was given intraperitoneally when required. All stereotactic coordinates were relative to bregma with the skull surface flat, according to Paxinos and Watson^36^. Thirteen small holes were drilled in the skull over the frontal area, primary motor area, primary somatosensory cortex, parietal cortex, and primary (secondary) visual cortex (regions potentially involved in SSVEP generation), and the temporal muscle was incised and drilled vertically to skull surface flat. Stainless-steel screw electrodes (diameter, 200 μm) were implanted in the drill holes, with the reference position at cerebrum (Cb), which exhibits lower activity compared to other brain sites^37^,^38^. The 13-electrode montage is shown in Fig. 4.

After the surgeries, all the rats recovered for one week in individual cages with a 12:12 h light: dark cycle (lights on at 8:00 A.M daily). For each rat, after the one week recovery period, the SSVEP experiment was carried out. During the experiments, the head of each rat was fixed using a specially designed box with a small hole through which the head can protrude but not move freely. Rats were injected with sodium pentobarbital (60 mg/kg body weight) for general anesthesia to further reduce unexpected artifacts induced by body movement, to exclude the effect of other higher level cognitive activity, and to provide a more stable stimulus during the whole experiment. SSVEP has been shown to be able to be evoked under such anesthesia state^3^,^14^.

Experiments were performed in a well-lit and shielded dark room. Before the circular stimulus, the data of a 5 min long control period was recorded for each rat. Next, rats were sequentially exposed to the 8 Hz low frequency stimulus, the 44 Hz middle frequency stimulus, and the 84 Hz high frequency stimulus provided by a LED with tunable frequencies (the duration time for each stimuli frequency was 2 min, and a 2 min rest was set before each frequency stimulus). The LED was fixed approximately 6 cm over the nose of the rat, with a 7 voltage fed to the LED for all the stimuli. EEG was recorded with a UEA-FZ amplifier (SYMPTO Company, Beijing, China) using compatible software developed by our lab (1000 Hz sampling rate), and was filtered using a 50 Hz notch filter and 0.1–120 Hz band pass filter. All recordings were stored on a hard disk (Lenovo Company, NewYork, USA) for further analysis. The samples with obvious artifacts were visually checked and abandoned. Five 3s-long data segments, that are free of artifacts, are selected from each of the four conditions for each rat to perform the following causality analysis. Details of data recording could be referred to the literature^14^.

### Double column model for SSVEP response

**Figure 5:**
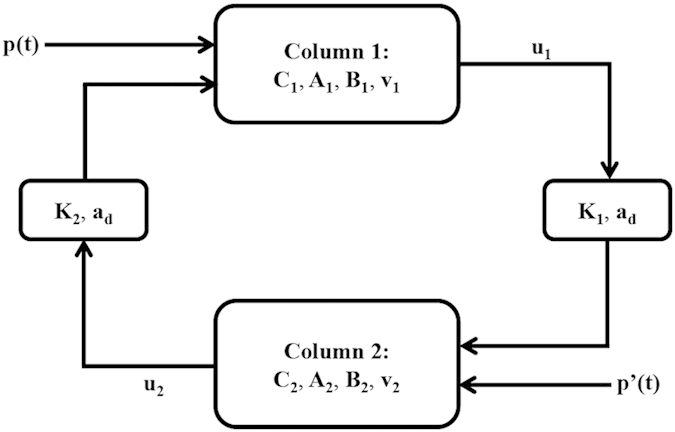
Double column model for SSVEP simulation. Column 1 represents the occipital lobe and Column 2 represents the frontal cortex.

The mechanisms of oscillation generation in the brain have been proven to be nonlinear^39^. Hence, nonlinear models should be used to describe cortical activity. The cortical column model, which is modeled by a population of ‘feedforward’ pyramidal cells, receiving inhibitory and excitatory feedback from local interneurons and excitatory input from the neighboring or more distant columns, is a kind of such nonlinear model^22^,^28^,^29^,^30^. The cortical column model has been used to simulate the oscillation of EEG waveforms. SSVEP is due to an interaction between two or more cortical areas^12^,^13^,^14^. Therefore, the more complex model is needed to more accurately simulate the actual brain mechanism of SSVEP. According to the introduction from Jansen and Rit, the double column model extended from the cortical column model was used to simulated the visual evoked potentials by describing the interactions between occipital and frontal regions^30^. As occipital and frontal areas are the two important cortical sources of the SSVEP, in current work, we used this double column model to simulate SSVEP waveforms, aiming to reveal the SSVEP mechanism based on this cortical model. The simplified double column model is depicted in Fig. 5.

Following Jansen and Rit, Column 1 and 2 represent the occipital and frontal area, respectively. C_1_ and C_2_ account for the total number of synapses established by interneurons onto the axons and dendrites of the neurons constituting the cortical column. C_1_ and C_2_ characterize the interaction between the pyramidal cells and the excitatory and inhibitory interneurons within the corresponding column. 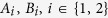 determine the maximum amplitude of the excitatory and inhibitory postsynaptic potential (EPSP and IPSP), respectively. 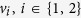 accounts for the firing thresholds of PSP. *a*_*d*_ is the constant for the information transfer delay between the two columns. 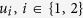 is the simulated waveform output at occipital and frontal area. *p*(*t*) and *p’*(*t*) are the external input for model at the two columns, where the white noise with amplitude distributed within 120–320 is usually fed to generate the spontaneous EEG activity. In current work, we used this double column model to simulate the SSVEP response by additionally introducing the circular pulse stimuli in the white noise series *p*(*t*). K_1_ and K_2_ are to delineate the coupling strength between the two columns, i.e., the information exchange. Besides those parameters in double column model, other parameters in the single double column were omitted and the detailed information of them could refer to the studies by Jansen *et al*.^29^,^30^. 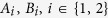, 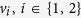 and *a*_*d*_ are the fundamental physiological parameters, and their values are physiologically determined and less influenced by outside stimuli. Therefore, in current work, we used the proposed values in Jansen and Rit for them, while mainly focused on the possible effect of coupling strength (i.e., C_1_, C_2_, K_1_, and K_2_) for the SSVEP stimulation. Generally, the coupling strength is directly correlated with the number of synapses established by interneurons onto the axons and dendrites of the neurons^26^,^29^,^30^,^40^. However, though the physical linkages are physiologically determined for the cortical column, it may assume that not all the synapses participate in the cognitive tasks^28^,^29^,^30^,^41^. That is to say, the different cognition task may have different number of synapses involved, reflecting the different information exchange within the single column (C_1_, C_2_) and between the double columns (K_1_, K_2_) as well. Especially, K_1_ and K_2_ may quantitatively reflect the directed information exchange between the two columns^30^.

### Inversing double column model based on PSO

Inspired from our previous work that different stimuli will have different evoked networks^14^,^16^, we evaluated if the related parameters of double column model (C_1_, C_2_, K_1_, and K_2_) could reflect the similar network linkages. In our recordings, the intracranial electrodes covered both frontal and occipital lobes. We selected the recordings at electrodes FrA_L and V1_L to serve as the reference signals at the two cortical areas. Based on the two reference signals, the concerned parameters (C_1_, C_2_, K_1_, and K_2_) were adjusted by PSO to generate the corresponding output u_1_ and u_2_, until the reference signals were well fitted by the two model outputs.

Compared to the traditional Newton based approaches, PSO is one of the evolutionary optimizations that are not necessary to know the expression between object function and concerned variables. PSO has been proved to be powerful to search for the global optima^42^,^43^,^44^,^45^. The standard PSO can be referred to the introduction from Shi *et al*.^44^. Aiming to find the suitable parameters to fit the recorded EEGs, the fitness object function for PSO is defined as,
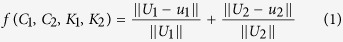
where U_1_ and U_2_ are the actual recorded EEGs at occipital (electrode V1_L) and frontal areas (electrode FrA_L), and u_1_ and u_2_ are the outputs of the two columns. According to the different frequency stimuli, the occipital input *p*(*t*) is generated by mixing the corresponding pulse signal into the white noise, and the frontal area input *p’*(*t*) is fed with the pure white noise. Except for C_1_, C_2_, K_1_, and K_2_, the model parameters like 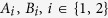, 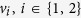 and *a*_*d*_ are set as the proposed values by Jansen and Rit^30^.

**Figure 6:**
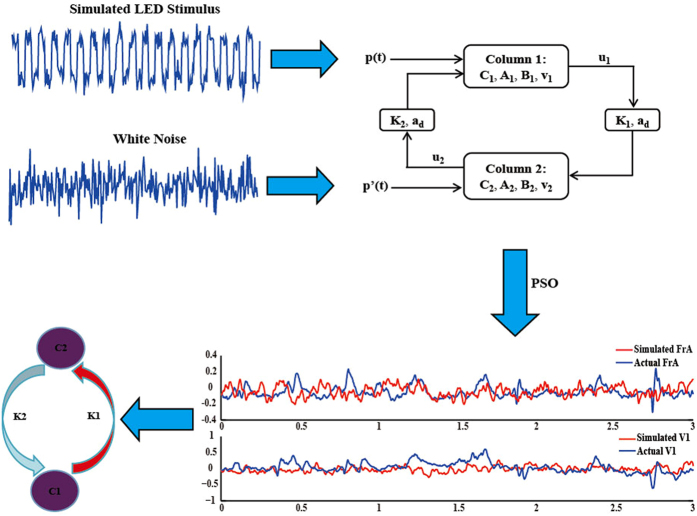
The flow chart for double column model based causality analysis.

When PSO was used to find the corresponding C_1_, C_2_, K_1_, and K_2_ for certain stimulus, the particles encoded the four parameters as 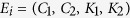 with *i* referring to the *i*th particle. Suppose the swarm consists of *Q* particles, the maximum generation number is *G*_max_, the termination error is *δ*, and the toleration iteration step number is *D*, The detailed values of these variables will be listed in the corresponding study. Following the proposed parameter ranges in Jansen and Rit^30^, the varying boundaries for K_1_, K_2_, C_1_, and C_2_ were 1500–3000, 100–600, 60–120, and 60–120, respectively. The PSO based model inversion for SSVEP response can be denoted as below flow chart,

The detailed procedure in Fig. 6 is depicted as follows:

Step 1. Particle initialization: Initialize each particle in swarm 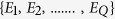 with 4 random values, where first component defines C_1_ within 60–120, the second component represents C_2_ distributed within 60–120, the third one is the information flow K_1_ within 1500–3000, and the fourth one is feedback flow K_2_ within 100–600. Initialize the velocity of the *Q* particles, 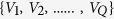, by setting the velocity of each particle, *V*_*i*_, with 4 random values having similar range as particle component. The *p*(*t*) is generated by adding 3 s long pulse waveform with 7.0 V amplitude to the white noise distributed within 120–320, and the pulse frequency is corresponding to the 3 frequency stimulus, while no pulse is added for the resting control state. *p’*(*t*) is the 3 s long white noise with amplitude distributed within 120–320. Let *P*_*i*_ be the *i*th particle position corresponding to the best fitness value of *i*th particle, and *P*_*g*_ represent the position of the best global fitness value ever achieved by all the particles.

Step 2. Evaluation of particle fitness: For the *i*th particle 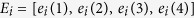, the current model parameters are defined by 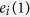, 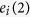, 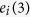, and 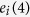. Based on the current model parameters, the corresponding outputs of double models are simulated, and the fitness *f*_*i*_ for *i*th particle is calculated following equation (1).

Step 3. Update the best position of each particle: Compare the fitness value *U*_*i*_ of the *i*th particle at current position (*E*_*i*_) with the ever achieved best fitness value by this particle at the position *P*_*i*_, if *U*_*i*_ is better, *P*_*i*_ will be replaced by the current position *E*_*i*_, else *P*_*i*_ will be remained. Here, 1 ≤ *i* ≤ *Q*.

Step 4. Update *P*_*g*_, the best position ever achieved by all the particles: Compare the renewed best fitness value of the *i*th particle at position *P*_*i*_ with the global optimal fitness value at *P*_*g*_, if the value at *P*_*i*_ is better, *P*_*g*_ will be replaced by *P*_*i*_, else *P*_*g*_ will be remained. Where 1 ≤ *i* ≤ *Q*.

Step 5. Update the velocity and position of each particle as,
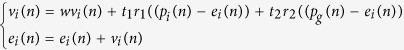
where 1 ≤ *n* ≤ 4, 1 ≤ *i* ≤ *Q*. 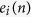 and 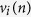 are the *n*th element and the *n*th velocity element of the *i*th particle, respectively; *w*, *t*_1_, and *t*_2_ are the same as those in the standard PSO.

Step 6. Judge the stopping criteria: If the number of generation is larger than the predefined number *G*_max_ or the decreasing of *P*_*g*_ has been less than the termination error *δ* in the continuous *D* iterations, the iterations will be stopped and the solution corresponding to the global optimal position (code chain) *P*_*g*_ is the final solution for the underdetermined system, else return to Step 2 and go on.

In the current study, the relative error (RE) is applied to evaluate the performance of model simulation, and the relative error is defined as,
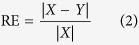
where *X* is the actual EEG signal and *Y* is the object signal simulated by PSO, 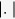 denotes the norm calculation for vector.

In the current work, the parameters of PSO were initialized as: The swarm size (population size) *Q* was 60; the number of generation *G*_max_ was 100; the inertia weight was updated as 
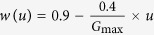
, where *u* was the current generation number; the velocity constants, *t*_1_ and *t*_2_ are both 2.0; the tolerance error *δ* is 10^−6^ and *D* is 20. After the estimation of model parameters for 5 segments of one rat, the corresponding C_1_, C_2_, K_1_, and K_2_ are then averaged across the 5 segments to achieve the model parameters for individual rat.

### Partial directed coherence

There are several causality analysis methods, i.e., GC, PDC, and DTF, etc., that can delineate the directed information flow for time series^21^,^23^. In current work, we adopted PDC to construct the directed network, aiming to reveal the information flow between visual cortex and frontal area for different frequency stimuli.

For each time series, the model coefficient can be calculated with the following equation,
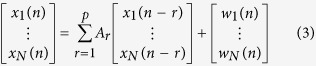

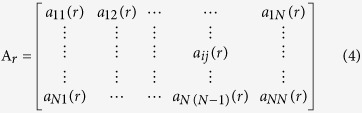
where *x* indicates the data vector, *w* indicates the multivariate independent white noise, *A*_*r*_ is the matrix of model coefficient which is estimated by the multivariate autoregressive (MVAR) model, *a*_*ij*_(*r*) represents the linear interaction effect of *x*_*j*_(*n − r*) onto *x*_*i*_(*n*). *p* represents the model order, which is estimated with Akaike Information Criterion (AIC)^46^,^47^ within range 5 to 20.

Then, PDC is the full multivariate spectral measure, which is recruited to assess the directed influences between any given pair of signals in a multivariate data set. PDC can be calculated as,
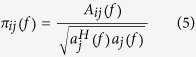
while
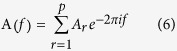
where *A*_*ij*_(* f*  ) is the *i*, *j*th element of *A*( *f*  ), *a*_*j*_(*f*  ) is the row vector of the matrix of model coefficient *A*(*f*).

For all *1* ≤ *j* ≤ *N*, the *π*_*ij*_(* f*  ) is subject to 
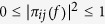
, meanwhile 
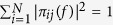
.

**Figure 7:**
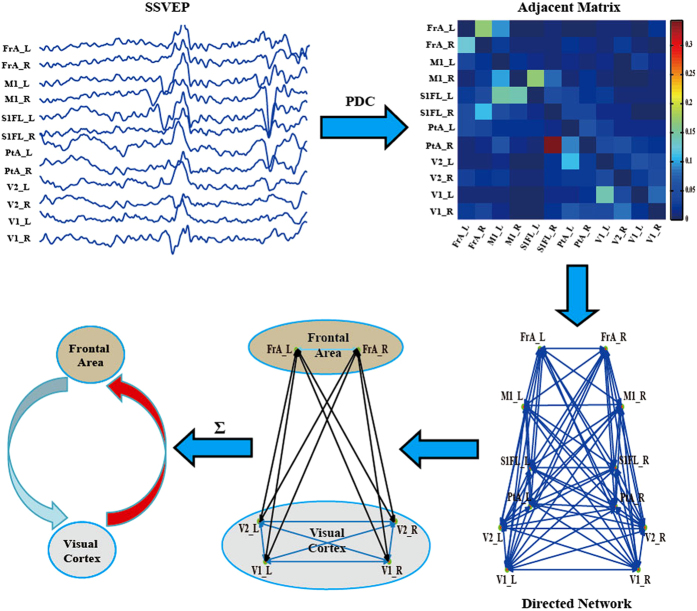
The flow chart for PDC based analysis.

The detailed scheme flow for the PDC based data-driven analysis is depicted in Fig. 7. The steps are listed as following:

Step 1. The 3 s long EEG segment is used to calculate PDC strength for the four conditions of each rat.

Step 2. Construction of directed networks. The directed flow strength between two electrodes is the average of PDC strength within frequency band 6–92 Hz. Based on the edge strengths for 12 electrodes, the directed network is constructed for the four conditions of each rat.

Step 3. Construction of sub-network between frontal area and visual cortex. Considering the positions of implanted electrodes, FrA_L and FrA_R are located in frontal area, while visual cortex covers V1_L, V1_R, V2_L, and V2_R. To build the information exchange between the concerned two areas, we select the corresponding directed information strengths among the six electrodes for further analysis.

Step 4. Calculation of overall information flow. The directed information from visual cortex to frontal area is the sum of directed PDC strength from electrodes in visual cortex (i.e., V1_L, V1_R, V2_L, and V2_R) to electrodes in the frontal area (i.e., FrA_L and FrA_R). On the contrary, the directed information from frontal area to visual cortex is the sum of directed PDC strength from electrodes in the visual cortex (i.e., V1_L, V1_R, V2_L, and V2_R) to electrodes in the frontal area (i.e., FrA_L and FrA_R). The information exchange within the frontal area is the sum of directed strengths between FrA_L and FrA_R, and the corresponding information exchange within visual area is the sum of directed strengths among the four electrodes (i.e., V1_L, V1_R, V2_L, and V2_R).

For each 3 s long segment of one rat under certain experiment condition, the corresponding information flow can be estimated with PDC. Then the PDC information flow of individual rat under certain condition is achieved by averaging linkages across the 5 segments.

## Additional Information

**How to cite this article**: Li, F. *et al*. The enhanced information flow from visual cortex to frontal area facilitates SSVEP response: evidence from model-driven and data-driven causality analysis. *Sci. Rep.* **5**, 14765; doi: 10.1038/srep14765 (2015).
